# Spinal Irritation

**Published:** 1882-02

**Authors:** 


### ORIGINAL TRANSLATIONS AND ABSTRACTS.

### SPINAL IRRITATION.

Prof. J. S. Jewell, of Chicago, in a recently published paper (*Journal of Nervous and Mental Disease*, October, 1881,) defines his views of the nature and treatment of what is often called, with more or less vagueness, spinal irritation. The more prominent characteristics of the affection are, in the first place, exaltation of the pain-sense in the nerves which enter the horizons of the spinal cord, which are the real seats of the affection. As a rule there are no alterations of sensibility, as numbness, tingling, prickling and other similar morbid sensations in the sphere of distribution of the nerves in question. There is not generally any marked diminution of the tactile sense. There is always exaltation of the pain-sense, which is more marked as the sensitive nerve trunks involved are shorter. The shorter the nerve trunk, the more irritable it seems to be; hence the chief external seat of the pain is greatest over the spinal column itself.

The hyperesthesia in spinal irritation is usually more marked upon a light than upon a heavier touch, especially if the latter is made gradually.

In spinal irritation there is no regular increase of temperature, disturbance of the circulation, or swelling in or beneath the morbidly sensitive region. There is no history of injury to the spinal column. Reflex excitability of the affected zones of the cord is rarely diminished; oftener increased.

There is rarely paralysis either of sensibility or motion in uncomplicated cases of spinal irritation. It rarely affects the entire length of the cord, but only certain zones, especially the lumbar, brachial or cervical. Nervous individuals are its subjects, as a rule. The pain is generally described as a "tired" or "fatigue" pain, relieved in a measure by rest in an easy posture, and made worse by exercise, though not to the same extent as in organic disease of the spinal column or dura mater.

In every case of true spinal irritation the chief seat of disease is in the sensitive tract of the spinal cord. It includes a nutritive lesion in which there is a loss of balance between waste and repair, an excess of the former over the latter. It is believed that, as in the case of the wasted muscle, or like leanness or loss of volume and weight in any given part, accompanied by a corresponding loss of energy or power, the same condition occurs in the exceedingly active and frequently overworked nerve mechanisms, especially those of the spinal cord. Any part of the spinal cord which is habitually over-excited or overworked, sooner or later may suffer not only a loss in volume and in power, but the process of wear and tear, when it has advanced to an extreme degree, even in a muscle, gives rise to irritation, the expression of which is at first a mere feeling of fatigue, but if the process of wear is carried further, fatigue graduates into pain.

Constantly recurring waste causes a more or less permanent lesion of nutrition, and consequently permanent fatigue and pain.

Circulatory disturbances in the spinal cord are not essential factors in the production of spinal irritation. Both congestion aud anemia are mere incidents in the course of the disorder. The fundamental condition is the lesion of nutrition. This agrees with all the facts, and when once fully understood points directly to the path of recovery.

There are two great clinical classes of cases of spinal irritation: those depending on over action and those due to over-excitation.

In the first class, the altitudes of the cord most likely to be affected are the lumbar, the brachial, and sub-occipital zones. In the author's experience a large number of cases of spinal irritation were found to be due to over-use of the legs in walking, standing and other occupations in which they are strenuously or persistently used for long periods; other cases were due to over-use of the arms, as in sewing, embroidery, painting, piano practice, &c., and finally a number of cases occur where the head is bent forward so as to put the muscles of the neck in a state of nearly unremitting tension. The conditions of action described imply a constant tide of innervation to the related muscles, and this again implies continuous fatiguing activity on the part of the spinal cord. How over-use of the cord, especially in persons of nervous and feeble constitution, may produce the lesion of nutrition described does not seem difficult to understand.

The group of cases depending upon over-excitation is not very well-defined. The horizons of the cord which may be the seat of irritation in this group are almost unlimited. The two principal levels of the cord affected are, however, the gastric and pelvic zones. In this class of cases the supposition is that some peripheral organ is the seat of the irritative disease. It is supposed that the sensory nerves which ramify in the diseased organ are, like its other structures, involved. It is further supposed that so long as the irritative disease exists in the organ, a more or less continuous tide of irritative "influences," is directed by way of its nerves into the corresponding altitude of the spinal cord.

Night and day, whether asleep or awake, an irritative influence enters the cord and contributes to the exhaustion and irritation of its related mechanisms. In this way it comes to pass that inflammatory or other irritative diseases of the uterus, ovaries, rectum, bladder, &c., lead, if persistent, to exhaustion and irritation of corresponding horizons of the cord. Hence, the all but uniform tenderness, exhaustion, pain, &c., in the lumbar and sacral regions of the spine in cases of irritative disease of the pelvic viscera. The gastric zone includes the stomach, liver and duodenum. Irritative disease involves their nerves and these become the channels of a disturbing influence which sooner or later exhausts and irritates the corresponding horizons of the spinal marrow. These horizons lie between the third and eighth dorsal vertebræ or in the inter-scapular region.

The spinal horizons which appear, clinically speaking, to stand in connection with the small intestine, are included between the eighth and eleventh dorsal and the second or third dorsal vertebræ. The spinal region corresponding to the pelvic zone, lies between the lower dorsal to the lower limits of the lumbar region, while disease of the rectum, especially about the anus, and of the cervix uteri finds its tender zone from the lower lumbar region to the coccyx. In the treatment of spinal irritation, the first principle is to put the part irritated at rest. This diminishes the waste of nutritive activity. The second is to supply good food and nerve tonics. Any irritative visceral disease existing must be combated by appropriate treatment. Special attention is called to two points in the treatment of this class of cases. Small doses of opium (one-twelfth to one-sixth of a grain of the aqueous extract, or a thirtieth to a tenth of a grain of muriate or bi-meconate of morphia combined with one-twentieth to one-eighth of a grain of extract of belladonna) twice or thrice a day constitute an important element in treatment by removing pain and securing rest.

The second point in treatment is the use of the electrical wire brush. The galvanic or induced current may be used. The sittings should not be oftener than once a day. Massage should be combined with the other plans of treatment.

